# Associations between Urinary Mercury/Cadmium Concentrations and Anthropometric Features in Korean Children

**DOI:** 10.3390/toxics12030175

**Published:** 2024-02-24

**Authors:** Min Won Shin, Hyo-Bin Kim, Ahreum Kwon, Mi Jung Park, Shin-Hye Kim

**Affiliations:** 1 Department of Pediatrics, Sanggye Paik Hospital, Inje University College of Medicine, Seoul 01757, Republic of Korea; s4255@paik.ac.kr (M.W.S.);; 2 Dr. Kwon’s Growth Clinic, Seoul 06506, Republic of Korea; 3 Dr. Park Mijung’s Child Growth Clinic, Seoul 05502, Republic of Korea

**Keywords:** obesity, heavy metals, pediatric population

## Abstract

Investigating the impact of urinary mercury and cadmium on anthropometric parameters in Korean children is crucial amid growing concerns about heavy metal exposure and childhood growth. Using data from the Korean National Environmental Health Survey (2015–2017), we assessed age- and sex-specific associations of urinary mercury and cadmium with height and body mass index (BMI) z-scores in 1458 children aged 3–5 (n = 571) and 6–11 years (n = 887). Overall, 5.0% had stunted height (3–5 years: 6.9%, 6–11 years: 3.8%), whereas older children exhibited higher overweight/obesity prevalence (29.2%) than younger ones did (22.2%). In 3–5-year-old boys, urinary mercury correlated negatively with height z-scores (*p* < 0.001), whereas in girls, urinary cadmium correlated positively (*p* = 0.015). Boys aged 6–11 years showed positive associations between mercury/cadmium levels and BMI z-scores (*p* = 0.012). Logistic regression indicated associations between urinary mercury and stunted height likelihood (*p* = 0.001) and between urinary cadmium and reduced overweight likelihood (*p* = 0.039) in 3–5-year-old boys. In boys aged 6–11 years, urinary cadmium levels were positively associated with overweight likelihood (*p* = 0.003). This study underscores the link between elevated urinary mercury, cadmium levels, and growth disruptions in Korean children, emphasizing the need for public health strategies for reducing childhood heavy metal exposure.

## 1. Introduction

Heavy metals, such as mercury and cadmium, are widespread environmental pollutants caused by human activities, such as mining, refining, and industrial processes [1]. These metals can contaminate the food chain by accumulating in biota and migrating into living organisms [2]. Human exposure to these metals occurs through ingestion, inhalation, and skin contact. Upon entering the body, heavy metals form strong organic complexes that irreversibly bind to essential ligands, interfering with carbohydrates, protein, and various energy metabolisms, ultimately leading to toxicity [3]. They can also induce oxidative stress, resulting in DNA damage, disruption of sulfhydryl homeostasis, and lipid peroxidation [3]. Research has suggested that pre- and postnatal mercury and cadmium exposure may cause irreversible neurological damage [4], impaired fetal growth [5], and metabolic bone diseases in infants and children [6]. 

Mercury has three toxicologically distinct forms: elemental mercury, inorganic mercury, and organic mercury. Industrial sources, such as mining, coal-fired power plant emissions, garbage incineration, and mercury-involved manufacturing, contribute to elemental and inorganic mercury pollution [7]. Despite its reduced use, elemental mercury is still found in products like thermometers and dental amalgams. Inorganic mercury compounds are present in products such as skin-lightening creams and disinfectants [7]. In contrast, methylmercury, the derivatives of which are the major source of organic mercury, is formed through the microbial transformation of mercury salts in aquatic environments and primarily enters the human body through seafood consumption [8]. The differential assessment of mercury exposure in the body is crucial: urinary mercury concentrations indicate exposure to elemental and inorganic mercury, as these forms are processed and excreted by the kidneys. Blood mercury levels, on the other hand, reflect methylmercury exposure. Methylmercury binds to red blood cells and circulates through the bloodstream, making blood tests a more accurate measure of this form of mercury [8]. 

Cadmium, similar to mercury, primarily emanates from activities such as mining, smelting, fossil fuel combustion, and waste incineration, leading to its airborne contamination [7]. It enters the food chain, accumulating in plants and animals, and is also found in higher amounts in cigarettes. Human exposure to cadmium occurs through eating, breathing, and skin contact, with the main sources being diet and smoking [8]. Both blood and urine are tested in assessing cadmium exposure; however, urine analysis is particularly critical for gauging long-term exposure. This is because cadmium accumulates over an extended period, primarily in the kidneys, and its gradual excretion is reliably indicated by its concentration in urine, reflecting the body’s cumulative exposure [7].

The existing research on the relationship between mercury or cadmium exposure and childhood growth has yielded inconsistent results. Some studies found no significant link between these exposures and childhood anthropometric measures [2], whereas others reported both negative [9,10] and positive impacts on growth [11,12]. Most research assessing the influence of mercury on infant and child growth has focused on blood mercury levels, mainly reflective of methylmercury exposure. Yet, there is a notable gap in research concerning the impact of elemental or inorganic mercury on children’s growth. Regarding cadmium exposure, most previous studies have measured blood cadmium levels, reflecting short-term or acute exposure [1,13]. It has been suggested that urinary tests, in contrast, are more appropriate for assessing long-term exposure to cadmium, as well as elemental and inorganic mercury [1].

Therefore, this study aimed to examine the association between urinary mercury and cadmium concentrations and anthropometric characteristics, such as height and body mass index (BMI), among Korean children using data from the Korean National Environmental Health Survey (KoNEHS).

## 2. Materials and Methods

### 2.1. Study Population

The study population included 1458 children aged 3–11 years who participated in the third cycle of KoNEHS between 2015 and 2017. The KoNEHS is a nationwide biomonitoring survey conducted to track harmful substance exposure trends in the Korean population. Representative samples of Korean children were recruited through kindergartens, childcare facilities, and educational institutions, which served as sampling units. Informed consent was obtained from both participants and their legal guardians. The survey collected quantitative data on environmental contaminant concentrations in blood and urine, along with information through a questionnaire on socioeconomic status, indoor and outdoor residential conditions pertaining to chemical exposure, medical histories, and lifestyle factors. All participants underwent measurements for urinary mercury and cadmium levels.

### 2.2. Ethical Approval

The KoNEHS adhered to the ethical standards and guidelines of the Declaration of Helsinki. All participants and their guardians provided written informed consent, ensuring their understanding and voluntary participation in the survey. The confidentiality and privacy of participants were rigorously protected throughout the study.

The survey design received approval from the Research Ethics Committee of the Korean National Institute of Environmental Research (NIER) under the ethical clearance numbers IRB No. NIER-2016-Br-003-01 and NIER-2016-BR-003-03. Furthermore, the current study’s protocol, including all procedures involving human participants, was thoroughly reviewed and approved by the Institutional Review Board of Inje University Sanggye Paik Hospital (Reference: IRB No. SGPAIK 2022-06-003). Given the nature of the study, the requirement for informed consent was waived by the approving board.

### 2.3. Assessment of Anthropometry

Qualified surveyors used a stadiometer and weighing scale to measure the heights and weights of the participants to the nearest 0.1 cm and 0.1 kg, respectively, while wearing light clothes without shoes. For this study, stunted height was defined as a height z-score below −1.0 standard deviation. BMI was calculated by dividing weight (kg) by the square of the height (meters squared). BMI categories were as follows: normal-weight (<85th percentile) and overweight (≥85th percentile), categorized on sex- and age-specific BMI percentile values derived from the 2017 Korean National Growth Charts for children and adolescents [14]. Height and BMI z-scores were determined using the lambda–mu–sigma formula. 

### 2.4. Measurement of Urinary Mercury and Cadmium

Urine samples were collected in the morning after a minimum of 8 h fasting. Samples were promptly delivered on the same day, stored, and refrigerated. Following transfer to a collection institution, the samples were processed within 24 h according to standard collection guidelines for each analysis item of biological samples. Subsequently, the samples were frozen and stored. Urinary mercury and cadmium concentrations were analyzed using a gold amalgamation direct mercury analyzer (DMA-80; Milestone, Shelton, CT, USA) and a graphite furnace atomic absorption spectrometer (240Z; Agilent, Santa Clara, CA, USA), respectively. The limits of detection for mercury and cadmium were 0.1 and 0.05 μg/L, respectively. All sample collection and analysis procedures were aligned with the protocols established by the NIER. 

The mercury content of the samples was analyzed by introducing them into a quartz tube within a DMA-80 device, where they underwent various heating stages to dry and thermally decompose the samples. The resulting decomposition products were then sent through a catalyst bed via continuous oxygen or air flow, which converted any mercury species present into elemental mercury. This elemental mercury was then captured in a gold amalgamator, and the mercury vapor released upon heating the amalgamator was quantified using atomic absorption spectrophotometry at a wavelength of 253.7 nm. The absorbance was directly proportional to the mercury concentration in the original sample.

For cadmium analysis, the samples were placed in a graphite tube and subjected to a controlled program of drying, ashing, and atomization within the furnace. The 240Z AA system was utilized, which applies a magnetic field during atomization using the Zeeman effect to enhance the accuracy of cadmium measurements. The absorption line was split for precise correction of non-specific absorption, allowing for a more precise measurement of cadmium concentration. The absorbance of cadmium was measured at a specific wavelength and its concentration was determined by calibration with standard solutions.

For internal quality assurance and control, standard reference materials were used, including a 1000 µg/g mercury standard in hydrocarbon oil (PerkinElmer, Waltham, MA, USA) and a 1000 µg/mL cadmium standard in 5% HNO_3_ (Agilent, Santa Clara, CA, USA). The analytical laboratories engaged in external quality control initiatives, such as G-EQUAS in Germany and proficiency testing administered by NIER, on a biannual basis. Additionally, regular assessments of quality assurance and quality control were conducted, including evaluating the linearity and slope of the calibration curve, detection limit, accuracy, and precision. Detailed information regarding the sampling and storage methods, analytical techniques, quality assurance, and control measures have been documented previously [15].

### 2.5. Covariates

Covariates were chosen based on insights from previous research, aiming to comprehensively address factors that might influence both heavy metal exposure and anthropometric outcomes. Standardized questionnaires completed by the children’s guardians provided data on various variables, including household income, dietary patterns, physical activity, and environmental factors. Family income, an indicator of socioeconomic status, was categorized into four ranges based on monthly earnings in Korean Won (KRW): <3 million, 3–5 million, 5–7 million, and >7 million KRW. The presence of environmental tobacco smoke exposure at home was assessed. Regular physical activity was defined as engaging in activities that induce perspiration and shortness of breath for at least 60 min per session on a minimum of 3 days per week [16]. Recognizing its relevance to both heavy metal exposure and body composition, grain intake was included as a covariate. It was categorized based on frequency: ≤twice and ≥three times per day. The study recognized meat and milk consumption as key indicators of nutrient-rich food intake. Meat and milk have been identified as pivotal sources of essential nutrients such as proteins, vitamins, and minerals, all vital for the proper growth and development of children [17]. The frequency of meat consumption was divided into ≤once, twice to thrice, and ≥ four times per week. Similarly, frequency of milk consumption was classified into ≤thrice, four to six times, and ≥seven times per week.

### 2.6. Adjustment of Urinary Dilution

Urine creatinine (Ucr) levels were analyzed using colorimetry (ADVIA 1800, Siemens, Tarrytown, NY, USA) by employing the Jaffe reaction method. For the adjustment of urinary dilution, we applied the covariate-adjusted standardization (CAS) method [18]. This approach first calculated the predicted Ucr level for each participant using linear regression models. These models included age, sex, and BMI as variables, with log-transformed Ucr as the outcome. Urinary heavy metal concentrations were presented as CAS-adjusted concentrations. The CAS heavy metal concentration was calculated using the equation:

[heavy metal concentration × (predicted Ucr/observed Ucr)]

### 2.7. Statistical Analysis

Statistical analyses adhered to the KoNEHS analytical guidelines and complex survey design factors, such as strata and sampling weights. Total urinary mercury and cadmium concentrations were distributed through selected percentile values. Geometric means (GM) with 95% confidence intervals (CIs) for urinary heavy metal concentrations were obtained by log-transforming the data.

CAS-applied total mercury and cadmium concentrations were categorized into quartiles, with quartile one as the reference. Linear regression models explored the relationship between urinary heavy metal concentration groups and height/BMI z-scores. In contrast, logistic regression models were used to evaluate the relationship between urinary heavy metal concentration and the prevalence of stunted height or overweight. Linear trend tests were conducted, considering urine heavy metal concentration groups as the continuous variables in the models. In the regression models, confounders, such as age, sex, family income, meat/milk consumption, and weekly walking time, were adjusted. Using stratified analysis, the effects of sex and age on the relationship between urinary heavy metal concentration groups and anthropometric parameters were investigated.

Generalized additive models (GAMs) with penalized splines were utilized to capture the non-linear associations between heavy metal exposure and anthropometric measures, accounting for sampling weights. All statistical analyses were performed using R software (version 4.0), with statistical significance set at a two-sided *p*-value < 0.05.

## 3. Results

### 3.1. General Characteristics of the Study Participants

Table 1 presents the study participants’ general characteristics. The study population consisted of 571 children aged 3–5 years and 887 children aged 6–11 years, with boys representing 51.6% of the total 1458 participants. The overall prevalence of stunted height was 5.0%, with a higher prevalence in the younger age group than in the older age group (6.9% vs. 3.8%). The older age group exhibited a significantly greater prevalence of overweight and obesity than the younger children did (29.2% vs. 22.2%). Most children (61.8%) reported a high grain intake, consuming it three or more times daily. The consumption of meat was less frequent, with 72.5% eating it once a week or less and only 22% consuming it two to three times per week. Approximately 45.5% of participants consumed milk more than seven times weekly. Regular physical activity was higher in the 6–11-year age group (42.4%) than in the 3–5-year age group (33.6%).

### 3.2. Distribution of Urinary Mercury and Cadmium Concentrations

Detection frequencies and urinary concentrations of mercury and cadmium are presented in Table S1. Mercury was found in 98.4% of the samples, whereas cadmium was detected in 91.5%. After CAS, the overall GM concentrations were 0.472 μg/L for mercury and 0.11 μg/L for cadmium. There was a significant age-related variation in urinary heavy metal concentrations. Children aged 3–5 years had higher GM mercury concentrations (0.642 μg/L) compared with those aged 6–11 years (0.431 μg/L, *p* < 0.05). In contrast, GM cadmium concentrations were higher in the 6–11 years age group (0.255 μg/L) versus the 3–5 years age group (0.139 μg/L, *p* < 0.05). Comparatively, Korean children had markedly higher mercury and cadmium urinary concentrations than did children in the United States, Canada, and Germany (Table S2) [19,20,21].

### 3.3. Association of Urinary Heavy Metal Concentrations with Height and BMI Z-Scores

Table 2 highlights significant age- and sex-dependent differences in the correlations between heavy metal concentrations and anthropometric indices. In boys aged 3–5 years, a significant negative correlation was observed between urinary mercury quartiles and height z-scores (*p*-trend < 0.001). This pattern was not evident in girls of the same age group. In contrast, a positive correlation between urinary cadmium quartiles and height z-scores was exclusively noted in girls aged 3–5 years (*p*-trend = 0.015). No significant correlations between heavy metal exposure and height were observed in the older age group. The analysis of BMI z-scores in the 6–11-year age group revealed positive associations between mercury and cadmium quartiles and BMI z-scores; however, this was limited to boys (*p*-trend = 0.012). In the younger age group (3–5 years), contrasting trends were evident: boys showed a modest negative correlation between urinary cadmium levels and BMI z-scores (*p*-trend = 0.064), whereas girls exhibited a significant positive correlation (*p*-trend = 0.026).

### 3.4. Associations between Urinary Heavy Metal Concentrations and Stunted Height or Overweight/Obesity

**Figure 1:**
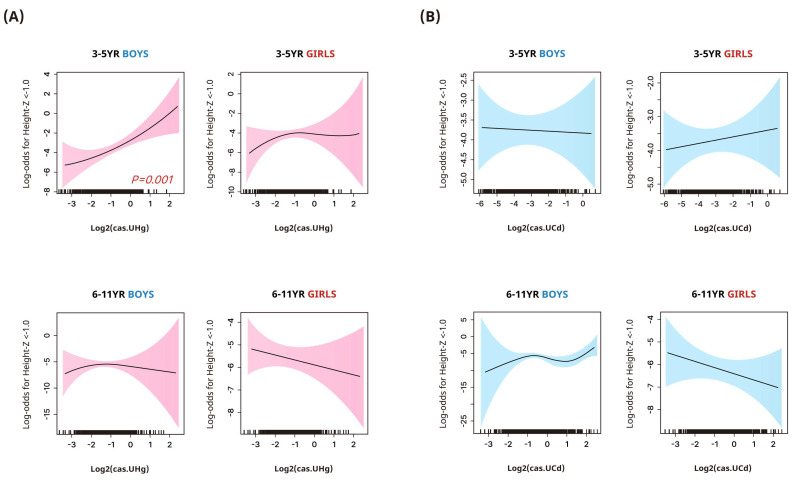
Smoothing plots of the associations between log2−transformed urinary mercury (**A**) and cadmium (**B**) concentrations and participants’ odds for stunted height (height z-score < −1.0) (95% confidence interval) in children. Urinary heavy metal concentrations were corrected for dilution using covariate−adjusted standardization and adjusted for age, family income, environmental tobacco smoke exposure at home, frequency of meat, milk, and grain intake, and regular physical activity.

Figure 1 and Table 3 present the adjusted odds ratios (95% CIs) for stunted height (z-score < −1.0) in relation to heavy metal concentrations stratified by sex and age group. In boys aged 3–5 years, there was a marginally significant increase in the odds of stunted height in the highest mercury quartile [4.4 (0.86, 22.55)] compared with the lowest (Table 3). This trend was more pronounced in the flexible dose–response analysis using GAMs (Figure 1, *p* = 0.001). No similar associations were observed in girls or children aged 6–11 years.

**Figure 2:**
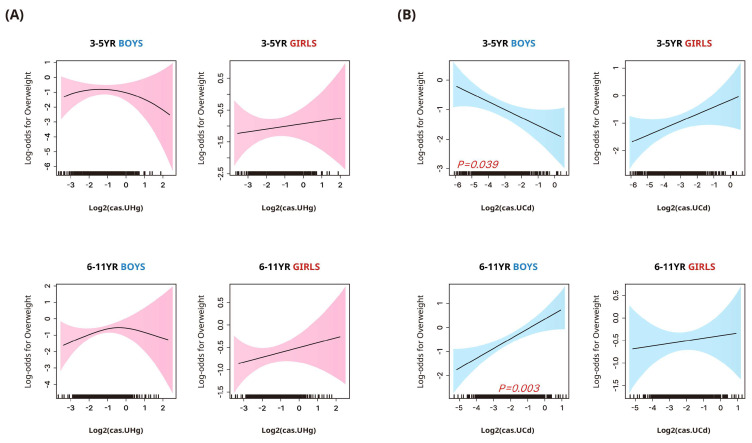
Smoothing plots of the associations between log2−transformed urinary mercury (**A**) and cadmium (**B**) concentrations and participants’ odds of being overweight (BMI > 85 percentile) (95% confidence interval) in children. Urinary heavy metal concentrations were corrected for dilution using covariate−adjusted standardization and adjusted for age, family income, environmental tobacco smoke exposure at home, frequency of meat, milk, and grain intake, and regular physical activity.

The adjusted odds ratios (95% CIs) for being overweight/obese according to the heavy metal concentrations stratified by sex and age group are presented in Figure 2 and Table 3. In boys aged 3–5 years, urinary cadmium concentrations were negatively associated with the likelihood of being overweight in the flexible dose–response analysis (Figure 2). However, this trend was not significant in the quartile-based analysis (Table 3). In the same age group, girls showed positive but non-significant associations between urinary cadmium and overweight likelihood. In boys aged 6–11 years, positive associations with the likelihood of being overweight/obese were noted in both quartile-based and flexible dose–response analyses, a pattern not observed in girls.

## 4. Discussion

This study investigated urinary mercury and cadmium concentrations in Korean children in relation to anthropometric indices, stratified by sex and age group, using nationally representative data. Our findings showed that in boys aged 3–5 years, higher urinary mercury concentrations were linked with increased odds of stunted height. Furthermore, in boys aged 6–11 years, elevated urinary cadmium concentrations correlated with a higher likelihood of overweight or obesity. Remarkably, urinary mercury and cadmium concentrations in Korean children were two to three times higher than those observed in children from the United States, Canada, and Germany. These findings emphasize the urgent need for government efforts to investigate and mitigate the potential risk factors for heavy metal exposure, aiming to reduce exposure in Korean children.

Urinary mercury concentrations are indicative of persistent environmental exposure to elemental and inorganic mercury, including from the atmosphere, soil, household dust, drinking water, and food [8]. Korea’s industrial activities, notably its energy sector and manufacturing industries, including gold mining and chlor-alkali plants, are major contributors to the country’s mercury emissions profile. In particular, the reliance on coal for energy production is responsible for releasing significant amounts of mercury into the atmosphere [22]. This is compounded by the broader context of Asia, responsible for nearly half of the world’s anthropogenic mercury emissions, largely due to industrial and energy-related activities [23]. Moreover, Korea’s geographic location exacerbates its mercury exposure due to atmospheric transport from regional sources across Asia, leading to elevated local mercury levels. This complex interplay between domestic industrial activities and regional environmental dynamics underscores the critical importance of Korea’s industrial sector in shaping the country’s mercury exposure landscape. 

In the present study, we found elevated urinary mercury concentrations in Korean children compared with Western children, echoing previous findings of elevated atmospheric mercury levels in Korean urban centers compared to North America and the Southern Hemisphere [24]. Recent research has also demonstrated a correlation between residential atmospheric heavy metal concentrations and urinary mercury concentrations in Korean adults, suggesting that urinary mercury levels could be an alternative indicator for atmospheric mercury exposure [25]. The dynamics of outdoor air can introduce airborne mercury to indoor environments, influencing indoor air quality and, subsequently, house dust heavy metal concentrations [26,27]. Young children are particularly vulnerable to mercury exposure from house dust due to their frequent hand-to-mouth and object-to-mouth behaviors, proximity to the ground, and tendency to spend more time on the floor [8]. In contrast, adults are primarily exposed to mercury via food consumption [8]. Therefore, higher urinary mercury concentrations in young children, compared with school-aged children, may reflect increased exposure to mercury-contaminated indoor dust, especially given the indoor-oriented activities of this age group. Despite the elevated concentrations of heavy metals detected in biological samples from the Korean population, research on heavy metals in outdoor/indoor air and household dust in Korea remains sparse. Our findings highlight the importance of expanded research in this area, focusing on heavy metal concentrations in indoor and outdoor environments, including household dust, in Korea.

In accordance with previous studies [28], our study revealed elevated urinary cadmium concentrations in Korean children relative to Western children. Although cigarette smoking is a known source of cadmium exposure, for non-smokers, dietary intake accounts for over 90% of cadmium exposure. In particular, rice consumption is a critical factor in East Asian diets, particularly in Korea [29]. This high rice consumption is a plausible explanation for the higher urinary cadmium levels in Korean children [28]. Additionally, our results suggest that older school-aged children have a higher urinary cadmium concentration than the younger age group. This may be attributed to increased grain consumption as they age and the bioaccumulation of cadmium, leading to a more pronounced cadmium body burden in these older children. Additionally, Korea’s industrial activities reportedly represent a potential source of the higher cadmium content in agricultural products through soil contamination [30]. Specifically, the country’s substantial production of refined cadmium, utilized in various industries including battery manufacturing, pigment production, and metal plating, contributes to environmental cadmium levels [31]. 

We found a negative linear association between urinary mercury levels and height in boys aged 3–5 years. This association adds to the growing body of evidence surrounding the impacts of mercury exposure on growth parameters. A recent systematic review reported that most cross-sectional and prospective studies identified a neutral association with birth length [32]. However, a subset of these studies indicated a negative correlation [32], suggesting a possible association between prenatal and postnatal mercury exposure and suboptimal growth outcomes during fetal development [10]. Meanwhile, research examining the connection between mercury exposure and childhood anthropometric indices is limited. A singular longitudinal study from Poland indicated that pre- and post-natal mercury exposure might hinder growth in early childhood [10]. However, a study from China [12] found no significant associations between blood mercury concentrations and height z-scores in children under six years. Similarly, Korean cross-sectional studies observed no significant associations between blood mercury concentrations and growth parameters in infants from birth to 24 months of age [33]. It is pivotal to note that many of these studies have based their observations on blood mercury levels, which predominantly measure exposure to organic forms of mercury, especially methylmercury [1]. Based on urinary mercury concentrations, our findings suggest a potential detrimental impact of inorganic mercury on children’s growth. Although the precise mechanism underpinning mercury’s impact on childhood growth is not fully understood, it is postulated that mercury’s endocrine-disrupting effects could play a pivotal role. Specifically, mercury may interfere with the hypothalamic–pituitary axis and thyroid function, which are integral endocrine regulators of chondrogenesis and linear growth [10]. Furthermore, the direct toxicity of mercury exposure on bone health, such as inhibition of osteoblast activity and impairment of ossification and bone growth, could partly explain our findings [6]. Comprehensive human studies and experimental research are essential to delve deeper into the possible repercussions of mercury exposure on pediatric growth and to elucidate the mechanisms at play.

Our study observed an inverse relationship between urinary cadmium concentrations and BMI z-scores, suggesting a decreased likelihood of overweight status in boys aged 3–5 years. This finding aligns with existing epidemiological evidence, posing that prenatal exposure to cadmium may lead to reduced adiposity during fetal development and later childhood [34]. However, the impact of cadmium exposure on anthropometric measures in children remains insufficiently investigated. Prior research, primarily concentrating on cadmium concentrations in blood or hair samples, has failed to delineate a definitive connection with children’s anthropometry [2,13,33]. Yet, two studies examining urinary cadmium levels in Bangladeshi children have identified negative correlations with height and body weight [9,35], corroborating our observations. The underlying mechanisms for these relationships are not entirely elucidated. Still, they are thought to involve an interplay of factors such as hormonal disruption, oxidative stress, renal impairment, and impaired nutrient absorption and transport [35].

Our study is of significant interest, as it is the first to indicate that urinary cadmium concentrations, a reliable measure of cumulative exposure, might be associated with obesity in elementary school-aged children. Some epidemiologic research, although not all [36,37], has revealed an association between cadmium exposure and obesity-related metabolic disorders in adults [38,39]. Supporting our findings, early-life cadmium exposure in animal models, including male mice and zebrafish, has been shown to increase adiposity [40,41]. Furthermore, experimental studies have shown that even low-dose cadmium exposure can influence adipocyte differentiation and adipokine expressions, potentially increasing the risk of metabolic syndrome and diabetes [42].

Our findings highlight that the effects of heavy metal exposure on anthropometric parameters may vary according to the children’s age, which could indicate different exposure windows or developmental phases affecting the body’s physiological response to heavy metal. Younger children are particularly susceptible to harmful substances due to their relatively higher consumption of air, food, and water than their body weight, more permeable skin, frequent hand-to-mouth and object-to-mouth behaviors, and higher surface-to-volume ratios. Additionally, the body’s metabolism of heavy metals changes with age, which may influence the observed age-specific correlations [43]. Multiple experimental and epidemiological studies have highlighted the importance of exposure timing, particularly in early life, for the health impacts of heavy metals [43]. Nevertheless, age-related differences in the impact of heavy metal exposure on childhood growth have been overlooked in previous epidemiological research. Our study found that the negative association between inorganic mercury exposure and height z-scores in boys was more evident in younger children than in elementary school-aged ones. Moreover, cadmium exposure negatively correlated with BMI z-scores in younger boys but exhibited a positive association in older boys. Our study’s results point out that the impacts of exposure to inorganic mercury and cadmium on anthropometric measures may rely on the timing of exposure relative to children’s developmental stages.

Numerous animal and human studies have demonstrated sex-dependent differences in the correlation between metal exposure and health outcomes. For example, male individuals appear to be more susceptible to kidney toxicity and neurotoxicity resulting from developmental mercury exposure than females [7,8]. However, most studies on the impact of heavy metals on prenatal or childhood body physiques have not addressed sex differences. The few that addressed this aspect reported inconclusive results [44,45]. Some experimental research has suggested that prenatal mercury exposure may negatively impact the body growth of male rodents, not females [46]. In line with this finding, our study revealed an association between inorganic mercury exposure and stunted growth only in boys. Regarding cadmium, the literature presents mixed findings about sex-based disparities in growth reductions following prenatal exposure [47]. Most studies probing the relationship between concurrent cadmium exposure and growth measurements found no significant links after adjusting for sex [2,13]. Of the two studies that implemented sex-stratified analyses, one found a negative correlation with height and weight only in girls [9], and the other found a negative correlation with BMI only in boys [35]. Our study found contrasting associations between urinary cadmium concentrations and BMI z-scores in boys and girls aged 3–5 years, which was not evident in the sex-adjusted analysis: a negative association in boys vs. a positive correlation in girls. Even though the sex-specific heavy metal–growth parameter associations cannot be clearly explained, accumulating evidence suggests sex-distinct epigenetic alterations due to heavy metal exposure in genes pivotal for early life growth and fat storage. For instance, in utero exposure to cadmium was linked to hypomethylation of cord-blood DNA responsible for organ and bone development in female individuals. However, in male individuals, changes predominantly occurred in genes associated with cell apoptosis [48]. Similarly, a recent study elucidated a sex-specific epigenetic alteration associated with mercury exposure, where modifications in DNA methylation patterns were identified in the *HDHD1* gene of cord tissue, correlating with mercury levels in cord blood. This phenomenon was exclusively observed in male individuals [49]. The *HDHD1* gene, mapped to the X chromosome, is pivotal in orchestrating diverse cellular processes, underscoring the complexity and specificity of heavy metal-induced genetic perturbations contingent upon sex. Further research is imperative to shed light on these sex-specific health effects of heavy metal exposure and their underlying mechanisms.

This study bears significant public health implications. It represents the first epidemiological research utilizing a nationwide biomonitoring survey of 1458 Korean children to illustrate an association between exposure to mercury and cadmium, stunted growth in childhood, and obesity. The urinary mercury and cadmium concentrations applied in this study have rarely been used in prior research examining the health implications of these metals, representing exposure to elemental/inorganic mercury and chronic cadmium, respectively. Moreover, we presented a correlation between heavy metal exposure and age- and sex-specific anthropometric indices.

Nevertheless, this study also has some limitations. Owing to its cross-sectional nature, we could not establish a definitive causal relationship. Moreover, the combined effect of exposure to complex mixtures of heavy metals might differ from the effects of individual heavy metals. Additionally, we were unable to adequately assess the participants’ status of minerals and micronutrients like iron, zinc, and calcium that might mitigate heavy metal toxicity. Another limitation is our methodology’s constraint in segregating the analysis between overweight and obesity due to the limited sample size, particularly after stratification by age group and sex. This limitation restricts our ability to explore the nuanced differences between these two conditions, which could provide valuable insights into their distinct epidemiological and clinical implications. Finally, we lacked access to data on the individuals’ birth size and their parental height.

## 5. Conclusions

In conclusion, we demonstrated that high exposure to mercury and cadmium is associated with stunted height and overweight among Korean children in an age- and sex-specific manner. These findings underscore the necessity for governmental actions to address primary heavy metal exposure sources and mitigate the associated health risks in Korean children.

## Figures and Tables

**Table 1:** General characteristics of the study population (n = 1458).

	Total	3–5 Years	6–11 Years	*p*-Value
n	1458	571	887	
Male (%)	51.6%	51.4%	51.7%	0.839
Age (year)	6.73 ± 0.22	4.01 ± 0.01	8.49 ± 0.11	<0.001
Height z-score	0.83 ± 0.04	0.74 ± 0.07	0.89 ± 0.05	0.115
Height z-score < −1.0	5.0%	6.9%	3.8%	0.020
BMI (kg/m^2^)	17.4 ± 0.1	16.1 ± 0.1	18.3 ± 0.1	<0.001
BMI z-score	0.29 ± 0.04	0.17 ± 0.08	0.36 ± 0.05	0.073
BMI status				0.023
Normal	73.5%	77.8%	70.8%	
Overweight/obesity	26.5%	22.2%	29.2%	
Family income (KRW)				0.752
<3,000,000	21.8%	21.8%	21.7%	
3,000,000–<5,000,000	38.2%	40.3%	36.8%	
5,000,000–<7,000,000	25.6%	24.1%	26.6%	
≥7,000,000	14.4%	13.8%	14.9%	
ETS exposure at home	6.4%	4.9%	7.4%	0.082
Regular physical activity	39.0%	33.6%	42.4%	0.006
Grain intake per day				0.098
≤2 times	38.2%	35.1%	40.2%	
≥3 times	61.8%	64.9%	59.8%	
Meat consumption per week				0.853
≤once	72.5%	71.9%	73.0%	
2–3 times	21.9%	22.1%	21.7%	
≥4 times	5.6%	6.0%	5.3%	
Milk consumption per week				0.480
≤3 times	28.6%	26.8%	29.8%	
4–6 times	26.0%	25.9%	26.1%	
≥7 times	45.4%	47.3%	44.1%	

Values are presented as percentage (%) or mean ± SE. ETS, exposure to environmental tobacco smoke; KRW, Korean Won.

**Table 2:** Adjusted changes (95% confidence intervals) in height or BMI z-scores with quartile group changes in urinary heavy metal concentrations by sex and age group.

	Urinary Heavy Metal Concentration				
	Quartile 1	Quartile 2	Quartile 3	Quartile 4	*p*-Trend
**Outcome: Height z-score**					
**Mercury**					
3–5 years					
Boys	Reference	−0.373 (−0.766, 0.021)	−0.467 (−0.854, −0.081)	−0.799 (−1.124, −0.475)	<0.001
Girls	Reference	−0.051 (−0.513, 0.412)	−0.336 (−0.825, 0.154)	−0.264 (−0.775, 0.247)	0.204
6–11 years					
Boys	Reference	−0.265 (−0.518, −0.012)	−0.198 (−0.451, 0.056)	−0.020 (−0.326, 0.286)	0.939
Girls	Reference	0.029 (−0.309, 0.367)	0.135 (−0.197, 0.466)	−0.229 (−0.564, 0.106)	0.344
**Cadmium**					
3–5 years					
Boys	Reference	−0.409 (−0.845, 0.027)	0.280 (−0.133, 0.693)	−0.005 (−0.366, 0.356)	0.298
Girls	Reference	0.338 (−0.106, 0.781)	0.670 (0.320, 1.020)	0.433 (0.008, 0.857)	0.015
6–11 years					
Boys	Reference	0.199 (−0.128, 0.527)	0.119 (−0.228, 0.466)	−0.073 (−0.405, 0.259)	0.532
Girls	Reference	0.328 (−0.019, 0.636)	0.11 (−0.165, 0.386)	0.019 (−0.316, 0.353)	0.806
**Outcome: BMI z-score**					
**Mercury**					
3–5 years					
Boys	Reference	−0.312 (−0.883, 0.259)	0.133 (−0.497, 0.763)	−0.013 (−0.542, 0.517)	0.652
Girls	Reference	−0.378 (−0.796, 0.039)	−0.023 (−0.486, 0.44)	0.082 (−0.421, 0.585)	0.486
6–11 years					
Boys	Reference	0.108 (−0.265, 0.482)	0.418 (0.036, 0.800)	0.413 (0.049, 0.777)	0.012
Girls	Reference	0.141 (−0.261, 0.543)	0.238 (−0.142, 0.618)	0.294 (−0.102, 0.689)	0.098
**Cadmium**					
3–5 years					
Boys	Reference	−0.453 (−0.962, 0.057)	−0.138 (−0.663, 0.386)	−0.676 (−1.236, −0.116)	0.064
Girls	Reference	0.084 (−0.355, 0.523)	0.145 (−0.337, 0.627)	0.519 (0.103, 0.936)	0.026
6–11 years					
Boys	Reference	−0.065 (−0.392, 0.263)	−0.032 (−0.386, 0.321)	0.477 (0.099, 0.855)	0.012
Girls	Reference	0.206 (−0.159, 0.571)	−0.18 (−0.527, 0.166)	0.371 (−0.006, 0.736)	0.266

Adjusted for age, family income, environmental tobacco smoke exposure at home, frequency of meat, milk, and grain intake, and regular physical activity. Urinary heavy metal concentrations were corrected for dilution using CAS before stratification into quartiles.

**Table 3:** Adjusted odds ratios (95% confidence interval) for having height z-score <−1.0 and being overweight/obese according to the quartile group changes in urinary heavy metal concentrations by sex and age group.

	Urinary Heavy Metal Concentration				
	Quartile 1	Quartile 2	Quartile 3	Quartile 4	*p*-Trend
**Outcome: Height z-score < −1.0**					
**Mercury**					
3–5 years					
Boys	1.00	2.04 (0.28, 14.92)	1.00 (0.11, 8.9)	4.4 (0.86, 22.55)	0.090
Girls	1.00	3.11 (0.70, 13.9)	3.84 (0.7, 21.05)	1.23 (0.18, 8.32)	0.872
6–11 years					
Boys	1.00	2.08 (0.4, 10.79)	1.7 (0.34, 8.62)	0.65 (0.11, 3.76)	0.589
Girls	1.00	0.68 (0.17, 2.62)	0.6 (0.16, 2.28)	1.07 (0.34, 3.36)	0.979
**Cadmium**					
3–5 years					
Boys	1.00	3.47 (0.74, 16.32)	1.31 (0.22, 7.86)	1.68 (0.30, 9.52)	0.946
Girls	1.00	0.64 (0.12, 3.42)	0.17 (0.02, 1.28)	1.84 (0.55, 6.13)	0.497
6–11 years					
Boys	1.00	4.19 (0.74, 23.56)	0.99 (0.15, 6.73)	1.11 (0.15, 8.53)	0.346
Girls	1.00	0.34 (0.11, 1.08)	0.41 (0.12, 1.48)	0.42 (0.12, 1.44)	0.175
**Outcome: Overweight/obesity**					
**Mercury**					
3–5 years					
Boys	1.00	0.83 (0.26, 2.68)	1.04 (0.38, 2.81)	0.96 (0.33, 2.83)	0.954
Girls	1.00	0.79 (0.26, 2.34)	1.27 (0.45, 3.6)	1.3 (0.39, 4.38)	0.555
6–11 years					
Boys	1.00	1.09 (0.43, 2.78)	1.41 (0.63, 3.12)	1.73 (0.76, 3.97)	0.161
Girls	1.00	1.23 (0.51, 3)	1.63 (0.64, 4.14)	1.7 (0.6, 4.83)	0.236
**Cadmium**					
3–5 years					
Boys	1.00	0.49 (0.2, 1.18)	0.78 (0.33, 1.85)	0.41 (0.13, 1.28)	0.205
Girls	1.00	0.86 (0.21, 3.58)	1.42 (0.47, 4.3)	1.85 (0.66, 5.2)	0.200
6–11 years					
Boys	1.00	0.77 (0.34, 1.76)	1.27 (0.64, 2.51)	2.44 (1.19, 5.00)	0.009
Girls	1.00	1.20 (0.64, 2.26)	0.86 (0.35, 2.14)	1.13 (0.51, 2.5)	0.985

Adjusted for age, family income, environmental tobacco smoke exposure at home, frequency of meat, milk, and grain intake, and regular physical activity. Urinary heavy metal concentrations were corrected for dilution using CAS before stratification into quartiles.

## Data Availability

The datasets used in this study, sourced from the KoNEHS, are not available for public access. Interested parties can request access to these data by contacting the Environmental Health Research Division at the National Institute of Environmental Research, which operates under the auspices of the Ministry of Environment in the Republic of Korea, via email at knehs@korea.kr. Please be aware that the authors of the current study are not authorized to distribute or share these data.

## Supplementary Materials

The following supporting information can be downloaded at https://www.mdpi.com/article/10.3390/toxics12030175/s1, Table S1: Geometric mean and selected percentiles of urinary mercury and cadmium concentrations (μg/L); Table S2: Comparison of urinary mercury and cadmium concentrations [μg/L, geometric mean (95% CI)] among the pediatric population by country.
