# Supplementary material for: Associations between Urinary Mercury/Cadmium Concentrations and Anthropometric Features in Korean Children
Source: Toxics. 2024 Feb 24;12(3):175. doi: 10.3390/toxics12030175 (PMC10974096; doi:10.3390/toxics12030175)
Supplement: Supplementary file 1 [file toxics-12-00175-s001.zip › toxics-2877819-supplementary.pdf]

Table S1. Geometric mean and selected percentiles of urinary mercury and cadmium concentrations (μg/L).

	N	%DF	GM (95% CI)	Selected percentiles				
				25th	50th	75th	90th	95th
**Mercury**								
Total	1,458	98.4	0.472 (0.446, 0.499)	0.310	0.459	0.690	1.028	1.278
Age								
3–5 years	571	97.9	0.642 (0.489, 0.601)*	0.364	0.534	0.833	1.169	1.498
6-11 years	887	98.8	0.431 (0.407, 0.457)	0.291	0.430	0.610	0.878	1.125
**Cadmium**								
Total	1,458	91.5	0.11 (0.10, 0.12)	0.031	0.120	0.329	0.616	0.937
Age								
3–5 years	571	81.3	0.139 (0.109, 0.177)	0.062	0.133	0.303	0.509	0.706
6–11 years	887	98.1	0.255 (0.235, 0.276)*	0.166	0.246	0.386	0.622	0.817

Concentrations were corrected for dilution using covariate-adjusted standardization.
*P < 0.001 for the difference in urinary heavy metal concentrations between age groups.
CI, confidence interval; DF, detection frequency; GM, geometric mean.

Table S2. Comparison of urinary mercury and cadmium concentrations [μg/L, geometric mean (95% CI)] among the pediatric population by country.

	**Korea (this study)**	**US [19]**	**Canada [20]**	**Germany [21]**
	KoNEHS (2015-2017)	NHANES (2017-2018)	CHMS (2016-2017)	GerES (2015-2017)
	3–11 years	3–12 years	3–11 years	3–10 years
	n = 1,458	n = 740	n = 1,091	n = 1,133
**Mercury (μg/L)**				
≤ 5 years	0.42 (0.37–0.48)	< 0.13[b]	< 0.16[b]	0.07 (–)[a]
≥ 6 years	0.39 (0.37–0.42)	< 0.13[b]	< 0.16[b]	0.07 (–)[a]
**Cadmium (μg/L)**				
≤ 5 years	0.11 (0.09–0.14)	< 0.036[b]	< 0.066[b]	0.05 (–)[a]
≥ 6 years	0.23 (0.21–0.26)	< 0.036[b]	< 0.066[b]	0.07 (–)[a]

[a]These concentrations were not obtained in the relevant study.

[b]Values presented as "<" refer to concentrations below the respective LOD (limit of detection).

KoNEHS, Korean National Environmental Health Survey; NHANES, National Health and Nutrition Examination Survey; CHMS, Canadian Health Measures Survey; GerES, German Environmental Survey
